# Referral rate and false-positive rates in a hearing screening program among high-risk newborns

**DOI:** 10.1007/s00405-023-07978-y

**Published:** 2023-05-08

**Authors:** Kruthika Thangavelu, Kyriakos Martakis, Silke Feldmann, Bernhard Roth, Ruth Lang-Roth

**Affiliations:** 1 Department of Otorhinolaryngology, Head and Neck Surgery, University Hospital Marburg, Philipps-Universität Marburg, Baldingerstrasse, 35043 Marburg, Germany; 2 Department of Pediatric Neurology, Social Pediatrics and Epileptology, Justus-Liebig-University Giessen and University Hospital Giessen, Feulgenstr. 10-12, 35392 Giessen, Germany; 3 Department of Pediatrics, Faculty of Medicine and University Hospital Cologne, Kerpener Strasse 62, 50937 Cologne, Germany; 4 Department of Otorhinolaryngology, Head and Neck Surgery, University of Cologne, Kerpener Strasse 62, 50937 Cologne, Germany; 5 Department of Neonatology, Faculty of Medicine and University Hospital Cologne, Kerpener Strasse 62, 50937 Cologne, Germany

**Keywords:** Hearing screening, Newborn, False-positivity, High-risk newborns, AABR

## Abstract

**Aim:**

More studies exploring referral rates and false-positive rates are needed to make hearing screening programs in newborns better and cost-effective. Our aim was to study the referral and false-positivity rates among high-risk newborns in our hearing screening program and to analyze the factors potentially associated with false-positive hearing screening test results.

**Methods:**

A retrospective cohort study was done among the newborns hospitalized at a university hospital from January 2009 to December 2014 that underwent hearing screening with a two-staged AABR screening protocol. Referral rates and false-positivity rates were calculated and possible risk factors for false-positivity were analyzed.

**Results:**

4512 newborns were screened for hearing loss in the neonatology department. The referral rate for the two-staged AABR-only screening was 3.8% with false-positivity being 2.9%. Our study showed that the higher the birthweight or gestational age of the newborn, the lower the odds of the hearing screening results being false-positive, and the higher the chronological age of the infant at the time of screening, the higher the odds of the results being false-positive. Our study did not show a clear association between the mode of delivery or gender and false-positivity.

**Conclusion:**

Among high-risk infants, prematurity and low-birthweight increased the rate of false-positivity in the hearing screening, and the chronological age at the time of the test seems to be significantly associated with false-positivity.

**Supplementary Information:**

The online version contains supplementary material available at 10.1007/s00405-023-07978-y.

## Introduction

Permanent congenital hearing impairment is an important public health problem across the world, affecting about 0.5–3 out of every 1000 newborn babies in the general population [1]. The World Health Organization estimates that about 34 million children live with hearing impairment as of 2021 [2]. Children born with hearing impairment suffer a significant detriment in their speech and language development resulting in poorer literacy skills and lower academic performance compared to normally hearing peers [3]. The implementation of universal newborn hearing screening in recent years has enabled these children to have timely access to interventions such as hearing aids, and cochlear implantation, which can in turn aid normal language development [4].

The current hearing screening programs have ample room for improvement. Continued efforts to make screening programs more cost-effective are underway in many countries. One of the key components of optimizing the success rate of the hearing screening programs is reducing false-positive results, which can in turn reduce referral rates in accordance with the benchmarks set by the Joint Committee on Infant Hearing [5]. Increased referral rate, particularly in high-risk newborns, is not only costly but also results in parental anxiety [6].

The two widely used tests in newborn hearing screening programs worldwide are transient-evoked otoacoustic emissions (TEOAE) and automated auditory brainstem response (AABR) [7]. These tests are either used alone or in combination based on regional guidelines and infrastructure in newborn hearing screening programs [7]. Several factors, including chronological age of the newborn at the time of screening and gestational age at birth, have been found to influence false-positivity rates in hearing screening programs [8, 9].

Studies have separately assessed risk factors causing hearing loss, risk factors for failed test results, and risk factors influencing false-positive results in hearing screening programs, using varied data collection and methodology. However, there is considerable overlap between these groups of factors. Clear definition of what false-positivity or referral rate constitutes is lacking, with studies using different endpoints of hearing screening programs. Several studies have assessed each risk factor separately and for either TEOAE tests or AABR tests, thus making it difficult to interpret clinically or generalize to the entire hearing screening program.

The aim of this study is to report the referral rate and false-positivity rate and assess the main factors associated with false-positive test result in a cohort of high-risk infants in a University Children’s Hospital in North-Rhine Germany.

## Materials and methods

### Newborn hearing screening in Germany

Newborns in Germany have been screened for hearing loss through the universal hearing screening program since 2009. Databases of screening details and results are handled by 16 regional hearing screening and tracking centers (TC), spread across 13 states in Germany [10]. In the federal state of North-Rhine-Westphalia, two TCs connect the regional birth facilities at various levels, from normal obstetric clinics to neonatal intensive care units, as well as the regional follow-up sub-centers with the TCs. In North-Rhine, the screening is a two-stage process combining both TEOAE and AABR tests. Healthy newborns are screened with TEOAE, and if they fail the TEOAE, they are screened with AABR. In contrast, the high-risk newborn babies are directly screened with AABR, and if they fail the initial tests, a repeat AABR is done.

### Study setting

The high-risk newborns were defined as newborns with one or more of the JCIH listed risk factors and were hospitalized in the neonatology department [5]. The newborns included in this study were all hospitalized in the Department of Neonatology of the University Hospital of Cologne over a 6-year period from January 2009 to December 2014, and thus, all of them belong to the high-risk category. According to the local screening program, all these babies should directly receive AABR test. However, due to the non-availability and long waiting times for AABR devices, TEOAE was done wherever possible in the wards. But regardless of the results of the TEOAE, all babies eventually underwent AABR because of their high-risk status.

The neonatology department in the University Hospital of Cologne includes three levels of care. Level 1 has the very sick newborns who are admitted in the neonatal intensive care unit (NICU), level 2 includes newborns who need intermediate-intensive care, and level 3 includes newborns that require hospitalization and treatment but not intensive care. Many neonates were hospitalized in two or all three of the care levels during their hospitalization according to their needs. The TEOAE and AABR test machines were of the model Echo-Screen Plus (Fischer-Zoth Diagnosesysteme GmbH, Germering, Germany). Hearing screening was mainly carried out by nurses or midwives at the hospital. The training, followed by the issuing of a certification, was usually done once with the option of regular re-training through refresher classes. The local tracking center is in charge of the training and the classes are offered up to three times a year.

### Identifying factors influencing false-positive rates

A list of factors that might be associated with false-positives in AABR is lacking in the literature. To form such a list, a literature search was conducted in PubMed for factors affecting false-positivity and/or referral rate in newborn hearing screening from 1990 to 2021 using the key words “false-positivity” OR “referral rate” AND “newborn hearing screening”. Both “false-positivity” and “referral rate” were used, since a lot of studies use these terms interchangeably. In total, 356 English-language articles were eligible for abstract screening of which 155 were duplicates. From the remaining, we identified 28 studies assessing factors affecting false-positive results in newborn hearing screening tests or programs (supplementary table).

### Data availability and calculating false-positive rates

The details of hearing screening of newborns included in this study were extracted retrospectively from the database of the TC in North-Rhine. The results of TEOAE and AABR tests, the follow-up of the newborns that ‘failed’ the screening program and thus referred to further diagnostics at the pediatric audiology department were extracted. The results of the diagnostics and final diagnosis at the pediatric audiology department were also retrieved. At the pediatric audiology department, the referred babies received clinical otoscopic examination, Brainstem Electric Response Audiometry (BERA), repeat TEOAE, and distortion product otoacoustic emissions (DPOAE) during spontaneous sleep or during melatonin-induced sleep. The data of the screening process from the first test until diagnosis at the pediatric audiology center were used to calculate results that were false-positive, true-negative, and true-positive. A true-positive result was defined as newborn babies that failed the screening tests and were also identified with hearing loss after the final diagnostics at the pediatric audiology department, while a false-positive result was defined as newborn babies that failed the screening tests, but a hearing loss was ruled out through testing in the pediatric audiology. The remaining babies that passed the test were defined as having a true-negative result.

The unique screening identification number of the newborn babies from our cohort was used to extract the information on the pre-defined set of factors from the neonatology database in the University Hospital of Cologne. All patient records and information were anonymized and de-identified prior to analysis.

### Statistical analysis

*T* tests and Chi-square tests were used to compare characteristics across the two groups of false-positive and true-negative test results. A univariate logistic regression was done to check for associations between various factors and false-positivity. A *p* value of less than 0.05 was considered statistically significant. All analyses were performed using Stata 14.0 (StataCorp, Texas, USA).

## Results

Among the 4512 newborns screened for hearing loss in the neonatology department, 1923 (42.6%) were female. The mean gestational age at birth for the study population was 35.5 ± 4.2 weeks with a median value of 36 weeks (min: 22, max: 43). The mean birth weight was found to be 2607 ± 940 g with a median value of 2630 g (min: 240, max: 5370). While 2828 (62.7%) newborns were admitted in the intensive care, 593 (13.1%) were in the intermediate care, and 1091 (24.2%) were in level three, while they needed hospitalization but not intensive care. The mean duration of hospital stay of the newborns in our population was 18.5 ± 29.4 days with a median value of 7 days (min: 1 max: 394).

**Fig. 1:**
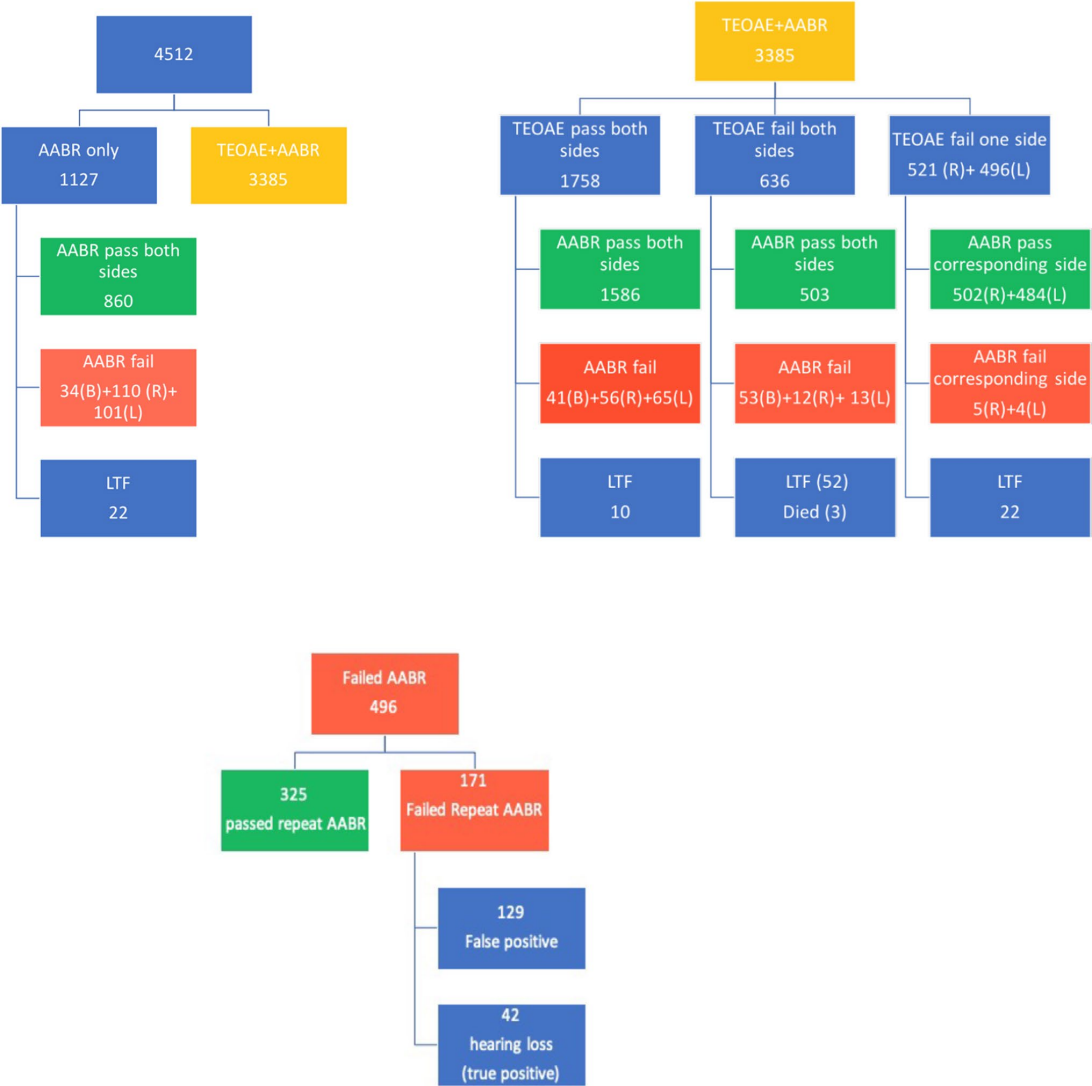
Hearing screening in 4512 high-risk newborns showing various stages and results (R right ear, L left ear, B both ears, LFT lost to follow-up)

1127 (25%) babies were screened with AABR, whereas 3385 (75%) were screened with both TEOAE and AABR. Among those screened with AABR, 860/1127 (76.3%) passed both sides and 245/1127 (21.7%) failed one or both sides. Among those screened with both TEOAE and AABR, 1758/3385 (51.9%) passed TEOAE both sides and went on to receive AABR, of which 1586 newborns also passed the AABR tests on both sides. 162 newborns failed AABR on one or both sides. A further 1653 of 3385 (48.8%) newborns failed TEOAE on one or both sides and received AABR, with 87 failing AABR on one or both sides and 1489 newborns passing AABR on both sides (Fig. 1). 106 (2.3%) babies were lost to follow up at various stages of the process; 3 babies died because of other comorbidities before the screening process was completed.

In total, 496 (11%) babies failed the AABR screening and received a repeat AABR. Of these, 171 (3.8%) also failed the repeat AABR test. They were referred to the pediatric audiology department (Fig. 1) and thus represent the referral rate of the study population. Among the 171 newborns, 83 failed AABR on both sides, 44 only on the right side, and 44 only on the left side.

Among the 171 babies that failed the hearing screening, 71 (1.6%) were identified with hearing loss at the pediatric audiology department (19 babies with sensorineural-, 33 with conductive- and 19 with combined hearing loss). Four of the 33 babies with conductive hearing loss were found to have atresia of the external auditory canal and were reclassified as permanent congenital hearing loss, resulting in a final total of 42 (0.9%) babies with permanent hearing loss. The rest of the 29 newborn infants with conductive hearing loss had transient hearing loss, either due to a middle-ear effusion or disorders of middle-ear ventilation [11]. In our cohort, 42 (0.9%) babies had a true-positive result, 129 (2.9%) had a false-positive result, and 4232 (93.8%) had a true-negative result, after excluding 109 (2.4%) babies that were lost to follow-up. The specificity of the hearing screening process was 97%.

### Risk factors influencing false-positivity

A list of the factors that might influence false-positivity was identified from the literature and is presented in Table 1. Data were available on five factors (chronological age of newborn at the time of hearing screening tests, gender, gestational age and weight at birth, and mode of delivery) and were derived from the neonatology database (Table 1) and analyzed in our cohort. The rest of the factors especially subjective environmental factors described in Table 1, such as ambient noise during testing, conditions of external ear, baby handling, bathing time, sleep/wake conditions, and maternal factors, could not be included because of the lack of data availability or of the poor quality of documentation in the neonatal database.

**Table 1:** List of possible factors influencing false-positivity in screening tests from literature

Factors	Tests tested for in the literature
1. Age at screening	TEOAE and AABR
2. Gestational age and birthweight	TEOAE and AABR
3. Gender (male)	TEOAE and AABR
4. Mode of delivery	TEOAE and AABR
5. Ambient noise during testing	TEOAE and AABR
6. Conditions of external ear, baby handling, bathing time, and sleep/wake conditions	TEOAE, AABR during sleep/wake conditions
7. Maternal factors: smoking, diabetes mellitus, and hypertension during pregnancy, analgesia with pethidine during labor, and epidural during caesarian section	TEOAE

Please refer to the supplementary table 1 for literature

Table 2 shows the prevalence of factors possibly influencing false-positivity included in this study, and among false-positives and among true-negatives. The prevalence of preterm birth was higher in the false-positive group than in the true-negative group (65% versus 52%; *p* = 0.003, difference 13%, CI 4.31–20.83) and this difference was found to be statistically significant. Whereas the prevalence of term birth was higher in the true-negative group than in the false-positive group (45% versus 34.9%; *p* = 0.021, difference 10.2%, CI 1.50–18.02). After categorizing the preterm births, extremely preterm (< 28 weeks) and very preterm (28 to < 32 weeks) also showed higher prevalence in the false-positive group.

**Table 2:** Prevalence of factors possibly influencing false-positivity among entire cohort, false-positives, and true-negatives

Factors		Entire cohort (*n* = 4512)	False-positives (FP) (*n* = 129)	True-negatives (TN) (*n* = 4232)^a^	Difference between FP and TN in % or means (Chi-square test, *p* value)
1. Gestational age at birth in weeks (125 missing values)	Term ≥ 37 weeks	2032 (46.3%)	45 (34.9%)	1910 (45.1%)	*p* = 0.0218 10.2 (1.50–18.02)
	Total preterm (< 37 weeks)	2355 (53.7%)	84 (65%)	2202 (52%)	*p* = 0.003 13 (4.31–20.83)
	(a) Extremely preterm (< 28 weeks)	252 (5.7%)	16 (12.4%)	228 (5.4%)	*p* = 0.000 7 (2.32–13.82)
	(b) Very preterm (28 to < 32 weeks)	368 (8.4%)	18 (14%)	342 (8.1%)	*p* = 0.016 6 (0.87–13.0)
	(c) Moderate-to-late preterm (32 to < 37 weeks)	1735 (39.6%)	50 (38.8%)	1632 (38.6%)	*p* > 0.05
2. Birth weight in grams (107 missing values)	Normal-birthweight (≥ 2500 g)	2403 (54.6%)	51 (39.5%)	2269 (53.6%)	*p* = 0.001 14.1 (5.34–22.25)
	Low-birthweight (< 2500 g)	2002 (45.5)	78 (60.5%)	1860 (44%)	*p* = 0.000 16.5 (7.74–24.65)
	(a) Extremely low-birthweight (< 1000 g)	278 (6.3%)	15 (11.6%)	255 (6%)	*p* = 0.009 5.6 (1.08–12.30)
	(b) Very-low-birthweight (1000 < 1500 g)	310 (7.0%)	17 (13.2%)	287 (6.8%)	*p* = 0.005 6.4 (1.54–13.35)
	(c) Moderately–low-birthweight (1500 < 2500 g)	1414 (32.1%)	46 (35.7%)	1318 (31.1%)	*p* > 0.05
3. Gender	Male	2589 (57.4%)	78 (60.5%)	2421 (57.1%)	*p* > 0.05
4. Chronological age of the infant at the time of screening	TEOAE	18.7 ± 31.2 (mean ± standard deviation)	39.7 ± 44.3	18.0 ± 30.3	*p* < 0.0001 21 (16.35–27.04)
	AABR	18.1 ± 31.0	31 ± 39.5	17.4 ± 30.0	*p* < 0.0001 13.6 (8.28–18.91)
5. Mode of delivery (292 missing values)	Caesarian	2377 (56.3%)	74 (57.4%)	2233 (52.8%)	*p* > 0.05
	Vaginal	1843 (43.7%)	53 (41.1%)	1723 (40.7%)	*p* > 0.05

Univariate logistics regression did not show any statistically significant association

^a^Excluding 109 lost to follow-up newborns

Similarly, the false-positive group had a higher proportion of low-birthweight babies in general, and extremely low-birthweight and very low-birthweight, compared to the true-negative group (60.5% versus 44%; *p* = 0.000, difference 16.5% CI 7.74–24.6) and this difference was statistically significant. The false-positive and true-negative groups were comparable in terms of prevalence of moderate-to-late preterm births (32 to < 37 weeks), and moderately low-birthweight babies.

The chronological age of the baby at the time of screening for TEOAE was much later in the false-positive group than among newborns who were true-negative (39.7 days ± 44.3 versus 18 days ± 30.3; *p* < 0.0001, difference 21 days CI 16.35–27.04) and this difference was found to be statistically significant. The same was true for the chronological age of infant at the time of screening for AABR (31 days ± 39.5 versus 17.4 days ± 30.0; *p* < 0.0001, difference 13.6 days, CI 8.28–18.91). The prevalence of Cesarean births in the false-positive group was higher than among the true-negative babies (57.4% versus 52.8%), but this difference was not statistically significant.

In the univariate analysis, gestational age at birth and birth weight were found to have statistically significant negative associations with false-positivity (Table 3); the higher the birthweight or gestational age, the lower was the odds of the screening results being false-positive. On the contrary, the chronological age of infant at the time of screening for both TEOAE and AABR was statistically significantly associated with false-positivity, showing that the older the infant at the time of screening, the higher the odds of being falsepositive. Gender and mode of delivery were not found to be associated with false-positive results. A multivariate logistic regression analysis including all the factors did not show any statistically significant associations.

**Table 3:** Univariate logistic regression showing association between various factors and false-positivity

Factors	Odds ratio	95% confidence interval	*p* value
Gender	0.87	0.61–1.25	0.461
Gestational age	0.92	0.89–0.95	0.000
Birth weight	0.69	0.57–0.83	0.000
Vaginal delivery	1.07	0.71–1.60	0.684
Date of screening TEOAE	1.0	1.00–1.01	0.000
Date of screening AABR	1.0	1.00–1.01	0.000

Detailed analysis of the chronological age of the newborns at the time of screening tests: The mean chronological age at screening with TEOAE was 18.7 ± 31.2 days and 18.1 ± 31.0 days with AABR tests. Among the babies who received both TEOAE and AABR, 3153 (93.1%; 3153/3385) received AABR and TEOAE on the same day, while 233 (6.9%) received AABR on a later day after TEOAE. The mean difference in days between TEOAE and AABR among newborns who received AABR (*n* = 233) on a later day was 18.3 ± 32.6 days (min 0, max 235).

Preterm babies (< 37 weeks) had higher mean chronological age at screening with TEOAE (28.8 ± 39.0 days) and AABR (27.3 ± 39.2 days). Similarly, low-birth-weight infants (< 2500 g) also received their tests later (mean chronological age at TEOAE of 32.8 ± 41.0 days and AABR of 30.5 ± 41.2 days).

The percentage of false-positivity for AABR based on the chronological age at the time of screening in days during the first month of life is shown in Table 4A. The lowest false-positivity rate for AABR tests was seen for infants who were screened within the first 5 days of life (1.5%) (Table 4A), irrespective of gestational age at birth and birth weight (Table 4B).

**Table 4:** False-positivity rates based on age at screening within 1st month of life: (A) AABR in total population, (B) AABR according to gestational age at birth, and (C) AABR according to birthweight (lowest false-positive rates marked in bold)

(A) AABR test	False-positive rate
Within 5 days	21/1430 **(1.5%)**
Within 6–10 days	31/1346 (2.3%)
Within 11–15 days	18/514 (3.5%)
Within 16–20 days	8/227 (3.5%)
Within 21–25 days	8/145 (5.5%)
Within 26–31 days	5/124 (4.0%)

(B) AABR test	False-positives in extremely preterm (< 28 weeks)	False-positives in very preterm (28 to < 32 weeks)	False-positives in moderate-to-late preterm (32 to < 37 weeks)	False-positives in term 37 weeks
Within 5 days	0/3	0/1	7/471 **(1.5%)**	14/835 **(1.7%)**
Within 6–10 days	0/1	0/3	14/522 (2.7%)	17/817 (2.1%)
Within 11–15 days	0/0	1/9 (11.1%)	9/349 (2.6%)	8/156 (5.1%)
Within 16–20 days	0/0	1/19 (5.3%)	6/156 (3.8%)	1/51 (2.0%)
Within 21–25 days	0/0	0/17	6/88 (6.8%)	2/40 (5.0%)
Within 26–31 days	0/0	2/37 (5.4%)	3/51 (5.9%)	0/36

(C) AABR test	False-positives in extremely low-birthweight (< 1000 g)	False-positives in very-low-birthweigh t (≥1000 < 1500 g)	False-positives in low-birthweight (1500 < 2500 g)	False-positives in normal-birthweight (≥ 2500 g)
Within 5 days	0/3	0/2	3/334 **(0.9%)**	18/986 **(1.8%)**
Within 6–10 days	0/1	0/3	12/392 (3.1%)	19/950 (2.0%)
Within 11–15 days	0/0	0/9	9/293 (3.1%)	9/212 (4.2%)
Within 16–20 days	0/0	0/15	5/141 (3.5%)	3/71 (4.2%)
Within 21–25 days	0/0	1/17 (5.9%)	5/85 (5.9%)	2/43 (4.7%)
Within 26–31 days	0/0	3/26 (11.5%)	2/63 (3.2%)	0/34

Table 4B shows the false-positivity rate for AABR, based on the chronological age of the newborn at the time of screening, categorized according to gestational age at birth. After categorization, the lowest false-positivity rate for AABR tests for moderate-to-late preterm infants (32 to < 37 weeks) and for term ≥ 37 weeks was still found among infants who were screened within the first 5 days of life. Such results could not be seen in the extremely preterm babies (< 28 weeks) and very preterm babies (28 to < 32 weeks), since few of them received the screening tests within the first month of life.

For example, all the extremely preterm newborns (< 28 weeks) except the 4 newborns in the Table 4B (4/252) were screened for AABR from the 40th day of life with the mean age of newborn for ABBR screening in the extremely preterm group being 107.9 ± 53.2 (min: 2 max 552) days. The mean chronological age of the newborn at the time of AABR screening was found to decrease with increasing gestational age at birth: very preterm (28 to < 32 weeks) at 45.2 ± 31.0 (min: 4 max 489) days, moderate-to-late preterm (32 to < 37 weeks) at 11.6 ± 14.5 (min: 1 max 184) days, and term ≥ 37 weeks at 8.4 ± 12.2 (min: 0 max 148) days.

Table 4C depicts a similar analysis categorized according to birth weight, showing similar findings. False-positivity rates for the first 5 days of life did not show any specific day or hour of life to be more optimal than the other (Table 5).

**Table 5:** False-positivity rates based on age at screening within first 5 days of life

AABR test	False-positives in entire cohort	False-positives in term (≥ 37 weeks)	False-positives in preterm (< 37 weeks)	False-positive in normal-birthweight (≥ 2500 g)	False-positive in low-birthweight (< 2500 g)
Within day 1 (≤ 24 h)	0/3 (0%)	0/1 (0%)	0 /0	0/1 (0%)	0/0
Within day 2 (25 to ≤ 48 h)	0/38 (0%)	0/35 (0%)	0 /1 (0%)	0/34 (0%)	0/3 (0%)
Within day 3 (49 to ≤ 72 h)	0/226 (0%)	0/129 (0%)	0/71 (0%)	0/152 (0%)	0/51 (0%)
Within day 4 (73 to ≤ 96 h)	8/727 (1.1%)	6/391 (1.5%)	2/258 (0.8%)	7/473 (1.5%)	1/185 (0.5%)
Within day 5 (97 to ≤ 120 h)	13/436 (3.0%)	8/279 (2.9%)	5/145 (3.4%)	11/326 (3.4%)	2/100 (2%)

## Discussion

Several protocols have been proposed for universal newborn hearing screening programs, using either TEOAE or AABR, or both. The two-stage process, first using TEOAE and a follow-up with AABR for newborns who failed TEOAE, has been shown to be the best option in the healthy newborn population [7, 12, 13] and has been accepted as a standard in Germany [10]. In case of high-risk newborns such as those admitted in intensive care, a two-stage AABR-only protocol may be a better option [14, 15]. Even though milder hearing loss is detected easier with TEOAE testing, a two-staged AABR-only protocol can capture auditory neuropathy spectrum disorder, which is characterized by an abnormal AABR in the presence of a normal TEOAE. The JCIH also recommends this protocol due to the higher incidence of auditory neuropathy spectrum disorders among neonatal intensive care babies compared to the healthy newborn population [16]. Although a combination of TEOAE and AABR was used in our study population, because babies’ high-risk statuses all were ultimately screened with AABR, irrespective of the TEOAE results, essentially making this a study of two-stage AABR hearing screening program for high-risk newborns.

Referral rates and false-positivity have been reported to be lower when only AABR was used instead of the two-step TEOAE and AABR protocol [8, 17], with our results showing an optimal 3.8% referral rate and 2.9% false-positivity rate. Even though referral rate is within the acceptable level (< 4%), the false-positive rate is still considered high [18]. In comparison to ours, a study of both well and NICU babies done in Spain showed a referral rate of 2.6% after the initial test with AABR and a referral rate of 0.32% after the second test with AABR [19]. This study also used a two-stage AABR protocol but included both well and NICU babies, in contrast to our study. The false-positivity rate was not reported as the outcome was only referred or ‘failed’ babies which most likely included both false-positive and true-positive results.

A Dutch study reported a similar referral rate for one-stage AABR of 3.1% among high-risk babies as part of implementation of the hearing screening program. A surprisingly higher referral rate of 9.3% was reported in 2011 as part of a quality control study of a two-stage AABR protocol to screen high-risk babies [20, 21]. A study conducted in Brazil in 2014 was one of the few in the literature that reported both referral- and false-positive rates for AABR tests among NICU babies. Two groups of babies were compared, one group underwent once only AABR test and the other underwent two-stage AABR, similar to our study. The study reported a referral rate of 18.6% and a false-positive rate of 62.2% for one-stage AABR group and a much lower referral rate of 4.1% for two-stage AABR group, which was comparable to our study. A false-positive rate of 12.5% was reported for two-stage AABR group, higher than our results [22]. The higher false-positive rate may be explained by the inclusion of conductive hearing loss as true-positive. In 2016, Pei-Chun Li et al. reported a referral rate of 2.8% among babies screened with AABR [23]. Recently Chang et al. [24] reported a referral rate of 6.8% among high-risk babies when using AABR only. Neither of these two studies reported the false-positive rates. The main aim of reducing the referral rate is to ensure that more babies are classified as true-negative and fewer receive false-positive results. We believe that most studies fail to report false-positivity rate due to unavailability of data and the extremely varied organization involved in hearing screening programs. Typically, the initial tests are done at different institutions, while the final diagnosis in referred babies happens at a specialized center with little or no cooperation between the different levels, thus making data collection very difficult.

Many reasons have been suggested for higher referral rate for two-stage TEOAE and AABR protocol when compared with two-stage AABR-only protocol. Inserting a probe into the ear canal during TEOAE can cause debris impaction. TEOAE test may lead to arousal of the newborn, which subsequently influence test results with AABR [25, 26]. The reason for initial TEOAE testing in our study, despite universal testing with AABR, was found to be due to non-availability of AABR as well as easier and shorter testing times of TEOAE. Our results show that among the newborns who received both TEOAE and AABR, the majority received both on the same day. Although TEOAEs can be conducted within a shorter test time in comparison to AABR, they cannot completely replace AABR. A two-stage TEOAE and AABR hearing screening protocol should be viewed critically for high-risk babies.

Several studies have been done to identify factors influencing false-positivity of hearing screening tests and protocols. Most of these studies were done for otoacoustic emissions (OAE) tests, and do not always report on the type of OAE tests used. Even fewer studies have analyzed factors affecting false-positivity for AABR (see supplementary file). Our study showed that preterm and low-birthweight babies showed increased false-positive rates, when compared to term and normal birthweight babies. This is in accordance with what has been reported in the literature. Delayed auditory maturation in these newborns may explain the results [27, 28].

The chronological age of the newborn at the time of screening appears to be an important factor influencing false-positivity regardless of the test or the protocol used. In our study, the later the AABR tests were done, the higher the chance of a false-positive result. In other words, the age of the newborn that had a false-positive test was considerably higher than those that had a true-negative test. Premature and preterm babies are typically screened much later. Preterm and low-birth-weight babies still had higher odds of getting a false-positive result. Several studies have suggested that performing the hearing screening tests, particularly TEOAE, after 48 hours of life decreases the referral rate and thus false-positivity considerably [12, 29, 30]. Possible reasons include temporary conductive hearing impairment in some newborns in the first hours of life, external auditory canal debris, and middle-ear amniotic fluid [29]. Very few studies have examined the timing of AABR testing [29, 31]. Our study did not show any specific results for those screened in the first 5 days of life, with the false-positivity rate being less than 1% for the first 4 days of life and increasing to 2.8% when screened on the 5th day of life. This may be attributed to two factors. First, we checked the false-positivity rate, while other studies assessed the referral rate or the incidence of test failure and second, our study population size, and thus, the number of newborn babies screened per day was relatively small.

Our study yielded lowest false-positivity rates within the first 5 days of life, with the false-positivity rate increasing as the days increased. This could be due to the fact that the sicker newborns are screened later, and with increasing chronological age, the babies exhibit a reduced need for natural sleep and tend to move during the test, increasing the chance of electromyogenic interference that occurs during the AABR test [32]. We believe that even though delaying the screening test till 48 hours of life as suggested in the literature is necessary for reducing false-positive tests, they should not be delayed longer than the fifth day of life. In reality though, screening within 5 days of life or even within the first month of life becomes difficult for extremely and very preterm (< 32 weeks) and extremely and very-low-birthweight (< 1500 g) infants.

Mode of delivery is another potential risk factor. In our study, the prevalence of Cesarean sections was higher in the false-positive group than the true-negative group, although logistic regression analysis did not show any association. Some studies have shown increased failure rates in hearing tests among babies born by Cesarean section [30, 33], while other studies have shown no association between mode of delivery and results of hearing screening tests [31, 34]. Most of these studies were performed for TEOAE; more studies are needed to assess associations of mode of delivery and AABR tests.

Many studies have been done evaluating environmental factors and maternal factors that might affect referral rate and/or false-positivity rate (supplementary table). Most of these studies analyze each factor separately and the documentation of such factors seems to be very subjective especially ambient noise during testing and baby handling, thus making their influence on false-positive rate or referral rate debatable.

Some studies have already discussed the possible ways to reduce false-positivity [18, 35]. Both studies offer performing an AABR before discharge as a solution to reducing false-positivity. Even though this is a viable solution for well babies, in a setup involving two-stage AABR protocol in high-risk newborns, this solutions becomes redundant. But recently, a study from Hungary reported using a third AABR test to reduce false-positive results following the two-stage AABR protocol [36]. Even though this could be a missing link in reducing false-positivity rate of two-stage AABR programs, implementing it on a larger scale might still not be cost-effective. A targeted third AABR could be considered, and for that, we believe that a closer examination of factors increasing false-positivity in specific populations is needed.

One of the limitations of our study is generalizability. Our study population consists of high-risk newborn babies who needed treatment in one of the neonatal care levels of a tertiary neonatal center; the generalizability of our findings should be done cautiously. Although the overall sample size was 4512 babies, the number screened per day was relatively small. For instance, only 18 newborns received AABR on the first day of life.

The newborns who passed the two-staged screening process were neither followed up over the years nor directly checked with detailed pediatric audiological diagnostics. Hence, the proportion of false negatives could not be estimated. This serves as a very important limitation of this study. Even if widespread implementation of long-term follow-up such as the school entry hearing screening in some parts of the UK is not economically viable, a targeted screening of high-risk newborns who passed the hearing screening might be a possible solution [37].

Another limitation is the known and unknown confounding factors when analyzing the factors associated with false-positivity. This might explain the loss of statistical significance in a multivariate analysis, making interpretations of combinations of risk factors difficult. Lastly, we were unable to include the environmental and maternal factors mentioned in the literature in our analysis because of lack of information. However, our study is the first to mention all possible factors from the literature together to the best of our knowledge. More studies are needed which document these factors together to better understand the importance of their role and discuss about the targeted third AABR test in specific groups.

## Conclusions

Among high-risk newborn babies, prematurity and low-birth weight increased the rate of false-positivity in a two-stage AABR hearing screening protocol, and the chronological age at the time of the test seems to be associated with false-positivity. Our study did not show a clear association between the mode of delivery and false-positivity in hearing screening. Among high-risk babies, we recommend performing a two-stage AABR-only newborn screening. We suggest further research focusing on referral rates and false-positivity for various screening protocols.

## Supplementary Information

Below is the link to the electronic supplementary material.

Supplementary file1 (DOCX 21 KB)

## Data Availability

The data that support the findings of this study are available from the authors but restrictions apply to the availability of these data, which were used under the license of the university hospital of Cologne and the medical ethics committee for the current study, and so are not publicly available for reasons of data protection and sensitivity. Data are, however, available from the authors upon reasonable request and with permission from the university hospital of Cologne and the medical ethics committee.
