# Supplementary material for: Referral rate and false-positive rates in a hearing screening program among high-risk newborns
Source: Eur Arch Otorhinolaryngol. 2023 May 8;280(10):4455–65. doi: 10.1007/s00405-023-07978-y (PMC10477105; doi:10.1007/s00405-023-07978-y)
Supplement: Supplementary file 1 — Supplementary file1 (DOCX 21 KB) [file 405_2023_7978_MOESM1_ESM.docx]

**Supplementary File:**

List of papers referring to factors influencing false positivity in screening tests:

|  | Factors | Paper | Year | No. of infants screened | Tests used |
| --- | --- | --- | --- | --- | --- |
| 1 | Age at the time of testing, Delivery mode | Auditory Brainstem Response Pass Rates Correlate with Newborn Hour of Life and Delivery Mode  Kelly et al  PMID: 33098840 | 2021 | 31984 | AABR |
| 2 | analgesia with pethidine during labour | Effect of analgesia with pethidine during labour on false positivity of newborn hearing screening test  Kadirogullari et al  PMID: 33882796 | 2021 | 143 | TOAE |
| 3 | maternal age, level of education,  parity, gravid, type of delivery, prenatal corticosteroid administration, prenatal complications and underlying diseases, amniotic fluid index (AFI), gestational age, the first and fifth minutes Agar scores, anthropometric measures, and history of hospitalization | Perinatal factors influencing the neonatal hearing screening results  Kaveh et al  PMID: 33407257 | 2021 | 181 | TOAE |
| 4 | Age at the time of testing | Referral rates for newborn hearing screening based on the test time  Chung et al  PMID: 31521889 | 2019 | 71,596 | OAE/AABR |
| 5 | Age at the time of testing/Discharge date | Does an early discharge of a newborn influence the success of the newborn hearing screening in developing countries? A hospital based study  Hrnčić et al  PMID: 30997785 | 2019 | 1167 | TEOAE |
| 6 | Normal or Caesarian section | The Effect of Mode of Delivery on Newborn Hearing Screening Results  Selis Gülseven Güven  PMID: 31049248 | 2019 | 2653 | TEOAE/  AABR |
| 7 | Age at the time of testing, Gender, race | Age and Other Factors Affecting the Outcome of AABR Screening in Neonates  Johnson et al  PMID: 29472244 | 2018 | 6817 | AABR |
| 8 | Preterm | Newborn hearing screening in prematurity: fate of screening failures and auditory maturation  Yang et al  PMID:27863036 | 2017 | 1375 NICU babies | TEOAE and AAABR |
| 9 | Ambient noice, Gentamicin | Effect of gentamicin and levels of ambient sound on hearing screening outcomes in the neonatal intensive care unit: A pilot study  Garinis et al  PMID:28483249 | 2017 | 82 infants (<37 weeks gestational age) | DPOAE and AABR |
| 10 | Amniotic fluid index, maternal tobacco, smoking | Newborn hearing screening failure and maternal factors during pregnancy  Schwarz et al  PMID: 29224768 | 2017 | 70/75 | OAE |
| 11 | mode of delivery (cesarean, vaginal) and hospital type (private, public) | The Effect of Mode of Delivery and Hospital Type on Newborn Hearing Screening Results Using Otoacoustic Emissions: Based on Screening Age  Farahani et al  PMID: 28239569 | 2017 | 2784 | TEAOE |
| 12 | Age at the time of testing | The universal newborn hearing screening program in a public hospital: The importance of the day of examination  Dimitriou et al  PMID: 27863649 | 2016 | 2494 | TEOAE |
| 13 | Preterm | Newborn hearing screening in Queensland 2009-2011: Comparison of hearing screening and diagnostic audiological assessment between term and preterm infants  Calcutt et al  PMID: 27521761 | 2016 | 175 911 | AABR |
| 14 | Age at the time of testing | Outcomes with OAE and AABR screening in the first 48 h--Implications for newborn hearing screening in developing countries  Van Dyk et al  PMID:25921072 | 2015 | 150 healthy newborns | TEOAE and AAABR |
| 15 | Ear coupler vs inserts | Improving newborn hearing screening: Are automated auditory brainstem response ear inserts an effective option?  Chan et al  PMID: 26412459 | 2015 | 167 | AABR |
| 16 | Age at the time of testing | Otoacoustic emissions in newborn hearing screening: a systematic review of the effects of different protocols on test outcomes  Akinpelu et al  PMID: 24613088 | 2014 | Systematic review | TEOAE and or DPOAE |
| 17 | Maternal HT, DM, Delivery mode, gender, birth weight, Age at the time of testing | The effect of maternal medications, hypertension/pre-eclamptic toxemia and diabetes mellitus on neonatal hearing screening  Ulanovsky et al  PMID:25417484 | 2014 | 2306 | OAE |
| 18 | Age at the time of testing | Delayed first otoacoustic emissions test decreases failure on neonatal hearing screening after caesarean delivery  Smolkin et al  PMID: 23363315 | 2013 | 560+566  Caesarian babies | OAE |
| 19 | Device used | Investigation of a significant increase in referrals during neonatal hearing screening: a comparison of Natus ALGO Portable and ALGO 3i  Hofmann et al  PMID:22212024 | 2012 |  |  |
| 20 | Post conceptional age in preterm | Influence of postconceptional age on universal newborn hearing screening in NICU-babies  Mueller-Mazzotta et al  PMID: 26557325 | 2012 | 634 NICU | AABR |
| 21 | Ambient noise | Ambient noise levels and infant hearing screening programs in developing countries: an observational report  Bolajoko O. Olusanya  PMID: 20557274 | 2010 | 11893 | TEOAE |
| 22 | Epidural with cesarean babies  Age of infant | The influence of epidural anesthesia on new-born hearing screening: A pilot study  Katijah Khoza-Shangase and Karin Joubert  PMID: 21430964 | 2011 | 20+20 | TEOAE+  AABR |
| 23 | Bathing time | Newborn hearing screening: the relation between bathing and the retesting rate  Marques et al  PMID: 18661011 | 2008 | 373+350 | TEOAE |
| 24 | Age at the time of testing | Effects of maturation on parameters used for pass/fail criteria in neonatal hearing screening programmes using evoked otoacoustic emissions  Sadri et al  PMID: 17389789 | 2007 | 19137 | TEOAE |
| 25 | Otitis Media with effusion | Failed newborn hearing screens as presentation for otitis media with effusion in the newborn population  Boone et al  PMID: 15733600 | 2004 | 76 | TEOAE |
| 26 | Age at the time of testing, population of infants being screened, the adequacy of probe fit, software options used, external ear conditions, screener training, and baby handling. | Maintaining acceptably low referral rates in TEOAE-based newborn hearing screening programs  Maxon et al  PMID:9397389 | 1997 |  | TEOAE |
| 27 | External ear debris | External and middle ear status related to evoked otoacoustic emission in neonates  Chang et al  PMID: 8435165 | 1993 | 41 | OAE |
| 28 | Sleep, awake-calm , awake active | Pediatric ABR screening: pass-fail rates in awake versus asleep neonates  McCall and Ferraro  PMID:1768867 | 1991 | 52 high risk | AABR |
